# m^6^A modification enhances the stability of *CDC25A* promotes tumorigenicity of esophagogastric junction adenocarcinoma via cell cycle

**DOI:** 10.7150/ijbs.98535

**Published:** 2024-08-06

**Authors:** Yongbo Pan, Huolun Feng, Jianlong Zhou, Wenxing Zhang, Yongfeng Liu, Jiabin Zheng, Junjiang Wang, Shan Gao, Yong Li

**Affiliations:** 1 Guangdong Cardiovascular Institute, Guangdong Provincial People's Hospital, Guangdong Academy of Medical Sciences, Guangzhou 510080, China.; 2 Department of Gastrointestinal Surgery, Department of General Surgery, Guangdong Provincial People's Hospital (Guangdong Academy of Medical Sciences), Southern Medical University, Guangzhou 510080, China.; 3 Zhongda Hospital, School of Life Sciences and Technology, Advanced Institute for Life and Health, Southeast University, Nanjing 210096, China.

**Keywords:** Adenocarcinoma of the esophagogastric junction, m^6^A, IGF2BP3, CDC25A, Cell cycle

## Abstract

*N*6-Methyladenosine (m^6^A) modification and its regulators play critical roles in human cancers, but their functions and regulatory mechanisms in adenocarcinoma of the esophagogastric junction (AEG) remain unclear. Here, we identified that IGF2BP3 is the most significantly up-regulated m^6^A regulator in AEG tumors versus paired normal adjacent tissues from the expression profile of m^6^A regulators in a large cohort of AEG patients. Silencing IGF2BP3 inhibits AEG progression *in vitro* and *in vivo*. By profiling transcriptome-wide targets of IGF2BP3 and the m^6^A methylome in AEG, we found that IGF2BP3-mediated stabilization and enhanced expression of m^6^A-modified targets, including targets of the cell cycle pathway, such as *CDC25A*, *CDK4*, and *E2F1*, are critical for AEG progression. Mechanistically, the increased m^6^A modification of *CDC25A* accelerates the G1-S transition. Clinically, up-regulated IGF2BP3, METTL3, and CDC25A show a strong positive correlation in TCGA pan-cancer, including AEG. In conclusion, our study highlights the role of post-transcriptional regulation in modulating AEG tumor progression and elucidates the functional importance of the m^6^A/IGF2BP3/CDC25A axis in AEG cells.

## Introduction

Adenocarcinoma of the esophagogastric junction (AEG) is defined as the adenocarcinoma that occurs in the esophagogastric junction within the range of 5 cm in both directions^1^. The incidence of AEG is increasing worldwide with unclear pathogenesis^2^, ^3^. Despite recent advances in genomic, transcriptomic, proteomic and phosphoproteomic profiling of large cohorts of AEG patients, and in multimodal treatments, AEG still lacks effective therapeutic targets and prognostic markers^4^-^7^. Therefore, it is imperative to understand the molecular mechanisms underlying AEG carcinogenesis and to identify potential prognostic markers and therapeutic targets.

*N*6-methyladenosine (m^6^A) modification plays a critical role in regulating mRNA fate in both physiology and pathology, including cancer^8^-^11^. This modification is installed by the m^6^A methyltransferase complex consisting of METTL3/METTL14/WTAP and other cofactors, and can be removed by the demethylases FTO and ALKBH5^12^-^14^. The functional effects of m^6^A on mRNA fate are mediated through m^6^A reader proteins, such as the YTH domain-containing proteins (YTHDF1/2/3) and insulin-like growth factor 2 mRNA-binding proteins (IGF2BP1/2/3)^15^-^17^. These m^6^A regulators have been shown to play critical roles in multiple cancers^10^, ^18^, ^19^. However, the role of m^6^A in regulating AEG tumor progression remains unexplored.

The function of IGF2BPs is well studied in multiple tumors^15^, ^20^-^22^, but its function and mechanism in AEG have not been elucidated. Here, we found that high IGF2BP3 promotes AEG progression by increasing *CDC25A* expression in an m^6^A dependent manner to accelerate the G1-S transition. Our study highlights the importance of post-transcriptional regulation in AEG tumor progression and the clinical significance of the m^6^A/IGF2BP3/CDC25A axis in AEG.

## Materials and Methods

### Patients and sample collection

Thirty treatment-naïve patients, who were newly diagnosed with AEG, were enrolled in this study. Prior to their surgeries, written informed consent was obtained from each patient, and fresh samples were collected post-surgery. The clinical characteristics of these patients are listed in Table S1.

### Plasmids

The wild-type (WT) IGF2BP3, METTL3, FTO, ALKBH5, and CDC25A, along with the mutant METTL3 (D377A, D395A, N539A, E532A), FTO (H231A/D233A, R316Q/R322Q), and ALKBH5 (H204A) expression plasmids were cloned into the pLVX-IRES-Neo vector. The dCas13b-ALKBH5/FTO and non-targeting gRNA plasmids were reported in our previous study^11^. Two gRNAs targeting the CDS of CDC25A were designed and cloned into the gRNA plasmid (Addgene plasmid #103854). The sequences of the gRNAs were:

gRNA1: 5′-CATACCGGCACATGCGGGGACCTCTCTCAGAAGAAAACTC-3′; gRNA2: 5′-GTTCATCCCACTGTGGCTCAGAGCAGCTTGACACGGTGCT-3′.

Mature antisense sequences of shRNA are listed in Table S2.

### Cell culture and transfection

Human AEG cell lines, including OE-19 (from a gastric cardia adenocarcinoma of a 72-year-old male patient) and SK-GT4 (from the primary tumor of an 89-year-old Caucasian man with an adenocarcinoma of the distal esophagus), were obtained from the Shanghai Cell Bank Type Culture Collection Committee (Shanghai, China). They were cultured in RPMI1640 medium (Gibco) with 10% fetal bovine serum (FBS) and 1% penicillin/streptomycin (Invitrogen). These cell lines were verified by short tandem repeat assays for their identification and tested negative for Mycoplasma contamination. The cells were cultured at 37^o^C in a humidified incubator with 5% CO_2_.

### RNA isolation and qRT-PCR

Total RNA was isolated using RNAiso Plus (Takara) and subjected to reverse transcription using the HiScript III All-in-One RT SuperMix Perfect for qPCR kit (Vazyme, China). qRT-PCR was performed using the Taq Pro Universal SYBR qPCR Master Mix kit (Vazyme, China) and analyzed with a QuantStudio 7 Flex Real-Time PCR System (Thermo Fisher). PCR results, recorded as Ct numbers, were normalized against *GAPDH*. The relative gene expression levels were analyzed using the 2^-ΔΔCT^ method. The primers used are listed in Table S3.

### Immunoblot

Proteins were extracted with lysis buffer (150 mM KCl, 10 mM HEPES pH 7.6, 2 mM EDTA, 0.5% NP-40, 0.5 mM DTT, 1:100 protease inhibitor cocktail), separated by SDS-PAGE, transferred onto PVDF membranes, blocked in 5% nonfat milk, and then blotted with specific antibodies (Table S4). Immunoblot results were normalized against GAPDH.

### Cell proliferation, migration, and colony formation assay

Cells were seeded in 96-well plates, with each well containing 1500-2000 cells in 100 μl of cell suspension. After a certain period in culture, cell viability was measured using the CellTiter-Glo Luminescent™ Cell Viability Assay (Promega). Migration assays were performed using transwell inserts with polyethylene terephthalate membranes (24-well inserts, 8.0 μm, Corning). 4×10^4^ cells in serum-free medium were added to the upper chamber, while 500 μl of medium supplemented with 10% FBS was added to the lower chamber to serve as a chemotactic agent. After incubation, cells that migrated through the filters were fixed with methanol and stained with crystal violet. The stained cells were destained with 33% acetic acid, and migration ability was assessed by measuring absorbance at 560 nm. For the colony formation assay, 1000 cells were cultured in a six-well plate at 37^o^C in a 5% CO_2_ humidified environment. After two weeks, cells were fixed, stained with crystal violet, and photographed.

### m^6^A-RIP

Total RNA was extracted using Trizol and treated with TURBO DNase (2 U/µl) (Invitrogen, AM2239), then fragmented by RNA fragmentation reagents (Thermo, AM8740). 100 μg purified RNA was incubated with A/G magnetic beads (Millipore) coated with 3 μg anti-m^6^A antibody (#202003, Synaptic Systems) at 4°C for 6 hours in 500 μl of NT2 buffer (200 mM NaCl, 50 mM HEPES pH 7.6, 2 mM EDTA, 0.05% NP-40, 0.5 mM DTT, 200 U ml^-1^ RNase inhibitor). 10 μg purified RNA was saved as an input sample. The beads were then washed eight times with 500 μl ice-cold NT2 buffer, and mixed with 1 ml TRIzol to save as the IP sample. Input and co-immunoprecipitated RNAs were recovered by TRIzol, extraction and analyzed by qPCR or RNA-seq.

### RIP

1×10^7^ cells were washed twice with 10 ml ice-cold PBS, then lysed in 400 μl lysis buffer with 400 U ml^-1^ RNase inhibitor, and supernatants were collected after centrifugation. Cell lysates were incubated with A/G magnetic beads (Millipore) coated with 3 μg anti-IGF2BP3 antibody (Proteintech, #14642-1-AP) at 4°C for 6 hours. 10% of the supernatant from the RIP lysates was saved as input. The following procedure was as described in the above m^6^A-RIP method.

### Luciferase reporter assay

To evaluate the effect of the 3′ UTR on CDC25A expression, the WT or mutant-m^6^A sites of the 3′ UTR of *CDC25A* were inserted behind the *firefly luciferase* (*F-Luc*) coding region. The pmirGLO-CDC25A-3′ UTR-WT and pmirGLO-CDC25A -3′ UTR-mutant were transfected into WT or METTL3/IGF2BP3 KD cells for 48 hours. The luciferase activities were detected using the Dual-Luciferase Reporter Assay System (Promega, USA). Renilla Luciferase (R-Luc) was used to normalize F-Luc activity.

### SELECT qRT-PCR

SELECT qRT-PCR was conducted as previously reported 23. Briefly, total RNA was quantified using the Qubit with Qubit™ RNA HS Assay Kit (Thermo Fisher Scientific). Total RNA (1000 ng) was mixed with 40 nM up primer, 40 nM down primer and 5 µM dNTPs in 17 µl of 1× CutSmart buffer (NEB). The RNA and primers were incubated at a temperature gradient: 90^o^C for 1 min, 80^o^C for 1 min, 70^o^C for 1 min, 60^o^C for 1 min, 50^o^C for 1 min and 40^o^C for 6 min. The mixture was then incubated with 3 µl of 0.01 U Bst 2.0 DNA polymerase, 0.5 U SplintR ligase, and 10 nM ATP at 40^o^C for 20 min, followed by denaturation at 80^o^C for 20 min. Subsequently, a 20 µl qPCR reaction was set up containing 2 µl of the final reaction mixture, 200 nM SELECT primers, and 1x Taq Pro Universal SYBR qPCR Master Mix (Vazyme, China). Primers for SELECT qPCR are listed in Supplementary Table S5. Ct values of samples were normalized to Control Ct values. All assays were performed in triplicate.

### RNA pulldown assay

OE-19 and SK-GT4 cells were lysed in lysis buffer containing 1% protease inhibitor cocktail. 2 nmol biotinylated RNA probes were incubated with 200 µg protein from cell extracts at 4^o^C for 4 hours in with 1× Protein-RNA Binding Buffer (20 mM Tris (pH 7.5), 50 mM NaCl, 2 mM MgCl_2_, 1% Tween-20). 30 μl of washed Pierce™ Streptavidin Magnetic Beads (Thermo Scientific™) were added to each binding reaction and further incubated at 4^o^C for 2 hours and then washed six times with Wash Buffer (20 mM Tris (pH 7.5), 10 mM NaCl, 0.1% Tween-20). The pull-down proteins were used for immunoblot analyses.

### mRNA stability

The stability of RNA in WT and METTL3 or IGF2BP3 KD cells was assessed by incubating cells with 5 μM actinomycin D (Act-D, Catalog #A9415, Sigma, USA). Cells were collected at the indicated times and RNA was isolated for qRT-PCR.

### Immunohistochemistry (IHC)

All slides were placed in a 60°C incubator for 20 min, de-paraffinized in xylene, and rehydrated in gradient ethanol. The slides were incubated with 3% hydrogen peroxide for 10 min, followed by antigen retrieval using 0.01 M citrate buffer (pH 6.0) for 30 min. After blocking with 5% BSA, slides were incubated overnight at 4°C with the relevant primary antibody. The secondary biotin-conjugated antibody was applied for 1 hour at room temperature. The IHC staining was visualized using diaminobenzidine reaction, counterstained with hematoxylin.

### RNA-seq

Total RNA was isolated from IGF2BP3 knockdown or control OE-19 cells using TRIzol. Poly(A) RNA was subsequently purified from 50-100 ng total RNA using the NEBNext Poly(A) mRNA Magnetic Isolation Module. The NEBNext Ultra Directional RNA Library Prep Kit (New England BioLabs) was used for library preparation. Each group was sequenced in triplicate.

### Linear amplification of cDNA ends and sequencing (LACE-seq)

IGF2BP3 LACE-seq was performed following the previously reported protocol^24^. Briefly, cells seeded in a 15-cm dish at 80-90% confluency were crosslinked by UV on ice at 400 mJ twice. 15 µg of IGF2BP3 antibody was used for RNA immunoprecipitation. The immunoprecipitated RNA was then fragmented by MNase, dephosphorylated, then subjected to reverse transcription, library preparation and deep sequencing.

### Sequencing data analysis

**For RNA-seq data**: RNA-seq was sequenced by Illumina NovaSeq 6000 with pair-end 150-bp read length. All clean reads were mapped to the human genome version hg38 by using hisat2^25^ with default settings. Read counts were calculated using featureCounts^26^. Differentially expressed genes between conditions were statistically assessed using the R/Bioconductor package DESeq2^27^.

**For RIP/meRIP-seq data**: Samples were sequenced by Illumina NovaSeq 6000 with pair-end 150-bp read length. The RIP-seq reads were mapped to the human genome version hg38 using Tophat2^28^ with default settings. RIP targets were called using MACS2^29^ with default settings. Motif analysis was performed using HOMER.

**For LACE-seq**: Clean reads were aligned to human pre-rRNA using Bowtie^30^, and the remaining unmapped reads were then aligned to the human genome version hg38 using Bowtie with parameters: -v 2 -m 10 -best -strata; -v 2 -k 10 -best -strata. Peaks were identified by Piranha^31^ with parameters: -s -b 20 -p 0.01. Motif analysis was performed using HOMER.

### *In vivo* animal assays

OE-19 cells with IGF2BP3 KD or CDC25A OE were injected subcutaneously (1 x 10^7^ cells/inoculum) into the flanks of five-week-old nude mice. Tumor formation/growth was assessed until the experimental endpoint, and tumor volume was calculated using the formula: (width)^2^ × length/2.

### Statistical analysis

Data were presented as the mean ± Standard Error of the Mean (SEM) or Standard Deviation (SD). Two-tailed Student's t tests were performed to assess the statistical significance of differences between groups. Spearman correlation was performed to analyze the correlation. *p* < 0.05 was considered statistically significant. All statistical analyses were performed using GraphPad Prism 8.0 or R software (version 4.2.2). *p < 0.05, **p < 0.01, ***p < 0.001; ns, not significant.

## Results

### Elevated IGF2BP3 expression correlates with poor prognosis of patients with AEG

**Figure 1:**
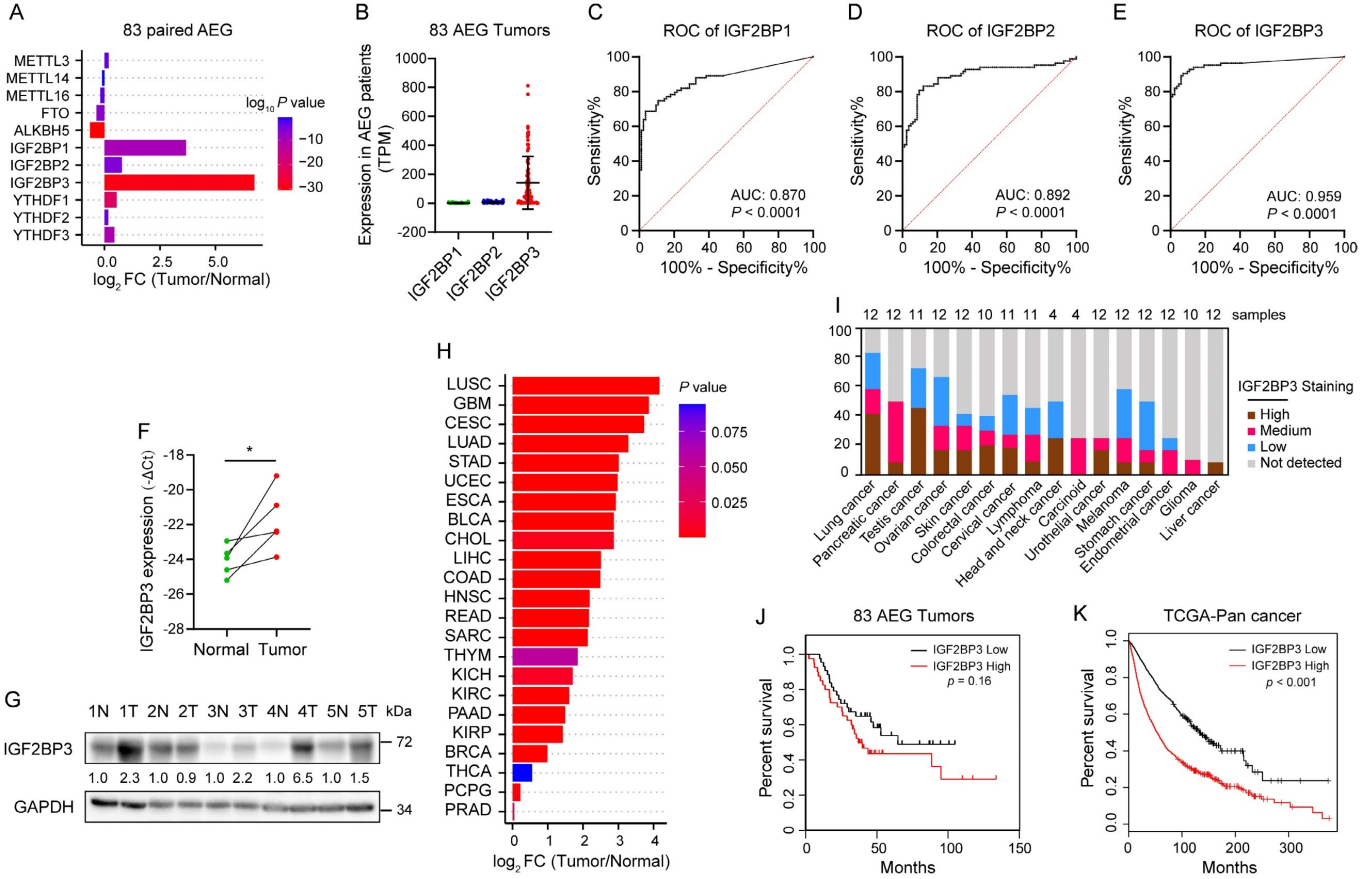
** Elevated IGF2BP3 expression correlates with poor prognosis of patients with AEG**. (A-B) Differential expression analysis of m^6^A modulators (A) and IGF2BPs (B) in 83 AEG tumors relative to adjacent normal tissue (PRJNA788008). (C-E) ROC analysis of IGF2BPs (C-D) in 83 AEG patients. FC, fold change. (F-G) mRNA and protein expression levels of IGF2BP3 in paired AEG tumors (T) and adjacent normal tissue (N) (n = 5). (H) Differential analysis of IGF2BP3 mRNA levels across 23 cancer types in TCGA datasets. (I) Expression levels of IGF2BP3 protein across 16 cancer types in HPA datasets. (J-K) Kaplan-Meier survival analysis of overall survival rates with low and high IGF2BP3 mRNA expression in AEG patients and TCGA Pan-cancer. *p < 0.05, **p < 0.01, and ***p < 0.001.

To explore the expression profile of m^6^A regulators in AEG, we analyzed published RNA-seq data from 83 AEG patients with paired tumor and adjacent normal tissues^6^. Compared with methyltransferase-like proteins (METTL3, METTL14 and METTL16), demethylases (FTO and ALKBH5), and YTHDF proteins (YTHDF1, YTHDF2 and YTHDF3), IGF2BP proteins had a higher fold change (FC) in their mRNA levels in AEG tumors versus paired normal adjacent tissues (Fig. 1A). Notably, among the IGF2BP proteins, IGF2BP3 had the highest FC (Fig. 1A) and mRNA level (Fig. 1B). Receiver operating characteristic (ROC) analysis also revealed that IGF2BP3 had the highest area under the curve (AUC) (Fig. 1C-E), which was chosen for further study. qRT-PCR and immunoblot also confirmed that the RNA and protein levels of IGF2BP3 were higher in 4 out of 5 (80%) AEG tumors than in their normal adjacent tissues (Fig. 1F-G). Furthermore, high expression of IGF2BP3 was observed in multiple cancers in The Cancer Genome Atlas (TCGA) and The Human Protein Atlas (HPA) datasets (Fig. 1H and I). Kaplan-Meier analysis showed that high IGF2BP3 expression was associated with poor survival prognosis in patients with AEG and TCGA pan-cancer (Fig. 1J and K). Taken together, these results revealed that IGF2BP3 is highly expressed in AEG and associated with poorer prognosis.

### IGF2BP3 plays oncogenic role in AEG

**Figure 2:**
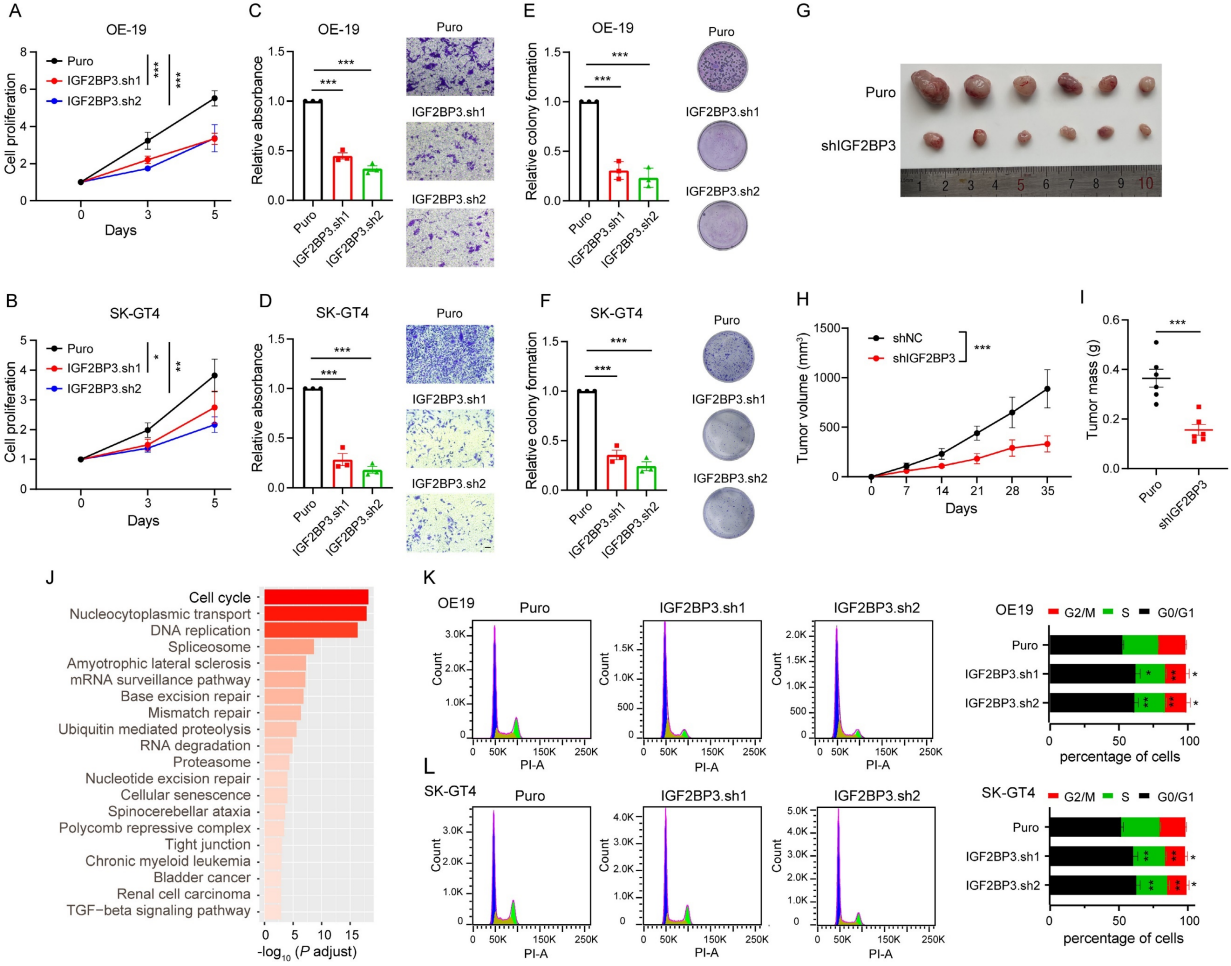
** IGF2BP3 plays oncogenic role in AEG**. (A-B) Cell proliferation assays assessing IGF2BP3 KD OE-19 (A) and SK-GT4 (B) cells. (C-D) Quantification (left) and representative micrographs (right) of IGF2BP3 KD OE-19 (C) and SK-GT4 (D) cells in non-coated Transwell assays. (E-F) Quantification (left) and representative images (right) of IGF2BP3 KD OE-19 (E) and SK-GT4 (F) cells in colony formation assay. (G-I) Effects of IGF2BP3 KD OE-19 cells on tumor size (G), tumor growth (H), and mass (I) in nude mice (n = 6). (J) KEGG analysis showing the top 20 significantly enriched pathways from down-regulated genes in IGF2BP3 KD OE-19 cells. (K-L) Flow cytometry analysis assays showing the percentage of IGF2BP3 KD OE-19 (K) and SK-GT4 (L) cells in sub-G0-G1, S, and G2-M cell-cycle phases (n = 3). Representative cell-cycle profiles (left) and quantification analysis (right) are shown. PI-A, propidium iodide-A. Scale bars, 20 µm. *p < 0.05, **p < 0.01, and ***p < 0.001.

We next determined the role of IGF2BP3 in AEG progression *in vitro* and *in vivo*. Cell proliferation and transwell assays showed that IGF2BP3 KD significantly decreased proliferation, migration, and colony formation of OE-19 and SK-GT4 cells (Fig. 2A-F). Furthermore, a subcutaneous tumorigenesis mouse model showed that IGF2BP3 KD inhibited *in vivo* xenograft tumor growth and mass of OE-19 cells (Fig. 2G-I), suggesting that IGF2BP3 KD can suppress tumor progression in AEG cancer.

To explore the functional pathways of IGF2BP3 in driving AEG, we conducted RNA-seq in IGF2BP3 KD OE-19 cells. The results of differential analysis showed 2966 significantly down-regulated and 2715 significantly up-regulated genes in IGF2BP3 KD cells (Fig. S1A-B). Kyoto Encyclopedia of Genes and Genomes (KEGG) pathway analysis revealed that the “Cell cycle” is the most significantly enriched signaling pathway among down-regulated genes (Fig. 2J), with a total of 75 genes associated with this pathway (Fig. S1C). Notably, 23 of these genes have been confirmed to be associated with the regulation of the G1-S transition (Fig. S1C and Table S6), of which 8 genes, including *CCNE2*, *CDC6*, *CDK4*, *CDC25A*, *CHEK2*, *E2F1*, *E2F3* and *WEE1* were selected for qPCR assays and were significantly reduced in the IGF2BP3 KD OE-19 cells (Fig. S1D). Indeed, flow cytometry assays showed that IGF2BP3 KD inhibited the G1-S transition in both OE-19 and SK-GT4 cells (Fig. 2K-L). Consistently, based on the median value of IGF2BP3 expression in the RNA-seq data of 83 AEG patients^6^, Gene Set Enrichment Analysis (GSEA) revealed that the “Cell cycle” signaling pathway was significantly enriched in the high-expression group (Fig. S2A-B). These results indicated that IGF2BP3 promoted AEG progression by accelerating the G1-S transition.

### IGF2BP3 KD globally down-regulates target gene expression

**Figure 3:**
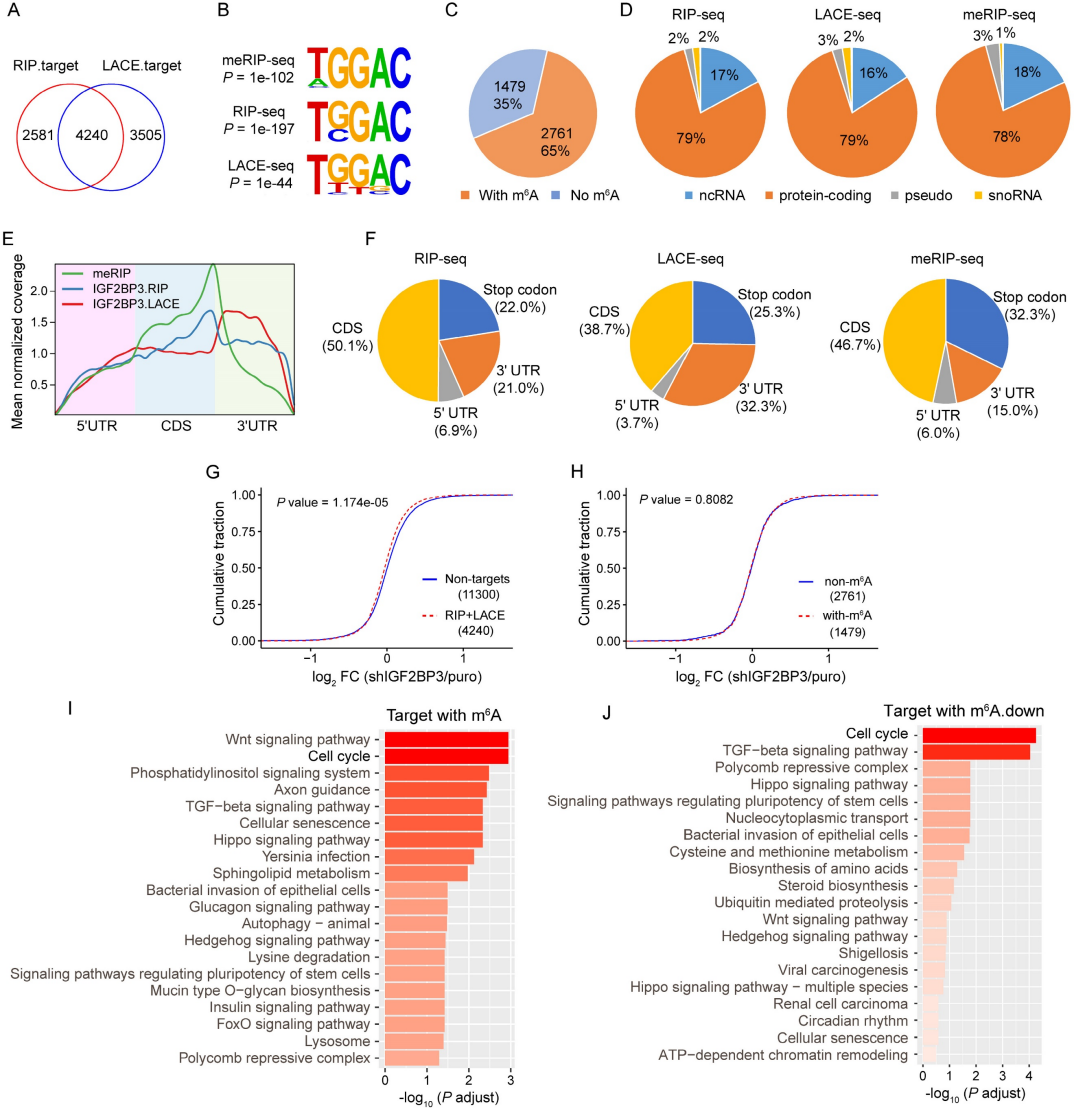
**IGF2BP3 KD globally down-regulates target gene expression.** (A) Overlap of IGF2BP3 target genes identified by RIP-seq and LACE-seq in OE-19 cells. (B) Top consensus sequences of IGF2BP3 binding sites and the m^6^A motif detected by HOMER motif analysis with meRIP-seq, RIP-seq and LACE-seq data. (C) Pie charts showing the numbers and percentages of IGF2BP3 high-confidence target genes containing m^6^A peaks. (D) Percentages of various RNA species in RIP-seq, LACE-seq, and meRIP-seq. (E) Metagene profiles showing enrichment of IGF2BP3 binding sites and m^6^A modifications across the mRNA transcriptome. CDS, coding sequence. UTR, untranslated region. (F) Distribution of IGF2BP3 binding peaks and m^6^A peaks within different gene regions. (G-H) Cumulative frequency of mRNA log_2_FC in non-target genes, IGF2BP3 high-confidence target genes (G), and IGF2BP3 high-confidence target genes with or without m^6^A (H) upon IGF2BP3 KD. *P* values were calculated using two-sided Wilcoxon and Mann-Whitney tests. (I-J) KEGG analysis showing the top 20 significantly enriched pathways from IGF2BP3 high-confidence target genes with m^6^A (I) and the overlapping genes from IGF2BP3 high-confidence target genes with m^6^A and downregulated genes in IGF2BP3 KD (J).

IGF2BP3 has been defined as an m^6^A reader recognizing and stabilizing a large repertoire of mRNA transcripts^15^. To understand the underlying mechanism of IGF2BP3 in AEG, we performed RIP-seq, LACE-seq and meRIP-seq in OE-19 cells. Firstly, we calculated the correlation between biological replicates, and confirmed good reproducibility of RIP-seq (r = 0.89, *P* < 2.2e-16), LACE-seq (r = 0.76, *P* < 2.2e-16), and meRIP-seq (r = 0.74, *P* < 2.2e-16, Fig. S3A). Sequencing purified RNA identified 6821 and 7745 potential target genes from RIP and LACE samples, respectively (Fig. S3B). Among them, 4240 genes overlapped with RIP and LACE-seq targets (Fig. 3A), which were considered as high-confidence targets. Further, meRIP-seq identified 7557 potential m^6^A modification genes (Fig. S3B). Both RIP and LACE targets preferentially bind to the 'UGGAC' consensus sequence containing the 'GGAC' m^6^A core motif^32^ (Fig. 3B), and 65% of the high confidence targets contain at least one m^6^A peak as detected by meRIP-seq (Fig. 3C). Moreover, most of the IGF2BP3 binding sites (79%) are located in protein-coding transcripts (Fig. 3D) and are highly enriched near stop codons and in 3′ UTRs, coinciding with the m^6^A distribution^33^ (Fig. 3E and F). We next integrated the meRIP-seq, RIP-seq, LACE-seq, and RNA-seq data. The global transcripts were grouped into non-targets and RIP+LACE targets (high confidence targets). IGF2BP3 KD globally inhibited the expression of its target gene (Fig. 3G), while IGF2BP3 KD had no significant effect on the expression of targets with or without m^6^A group (Fig. 3H).

To explore the major function of IGF2BP3 targets in AEG, we performed KEGG pathway enrichment analysis. Results showed that IGF2BP3 targets with m^6^A were significantly enriched in the “Cell cycle” signaling pathway (Fig. 3I). Furthermore, m^6^A down-regulated targets in IGF2BP3 KD were also significantly associated with the “Cell cycle” signaling pathway (Fig. 3J). Taken together, these results indicated that IGF2BP3 regulated the cell cycle in an m^6^A-dependent manner.

### IGF2BP3 stabilizes the *CDC25A* transcript in an m^6^A-dependent manner

**Figure 4:**
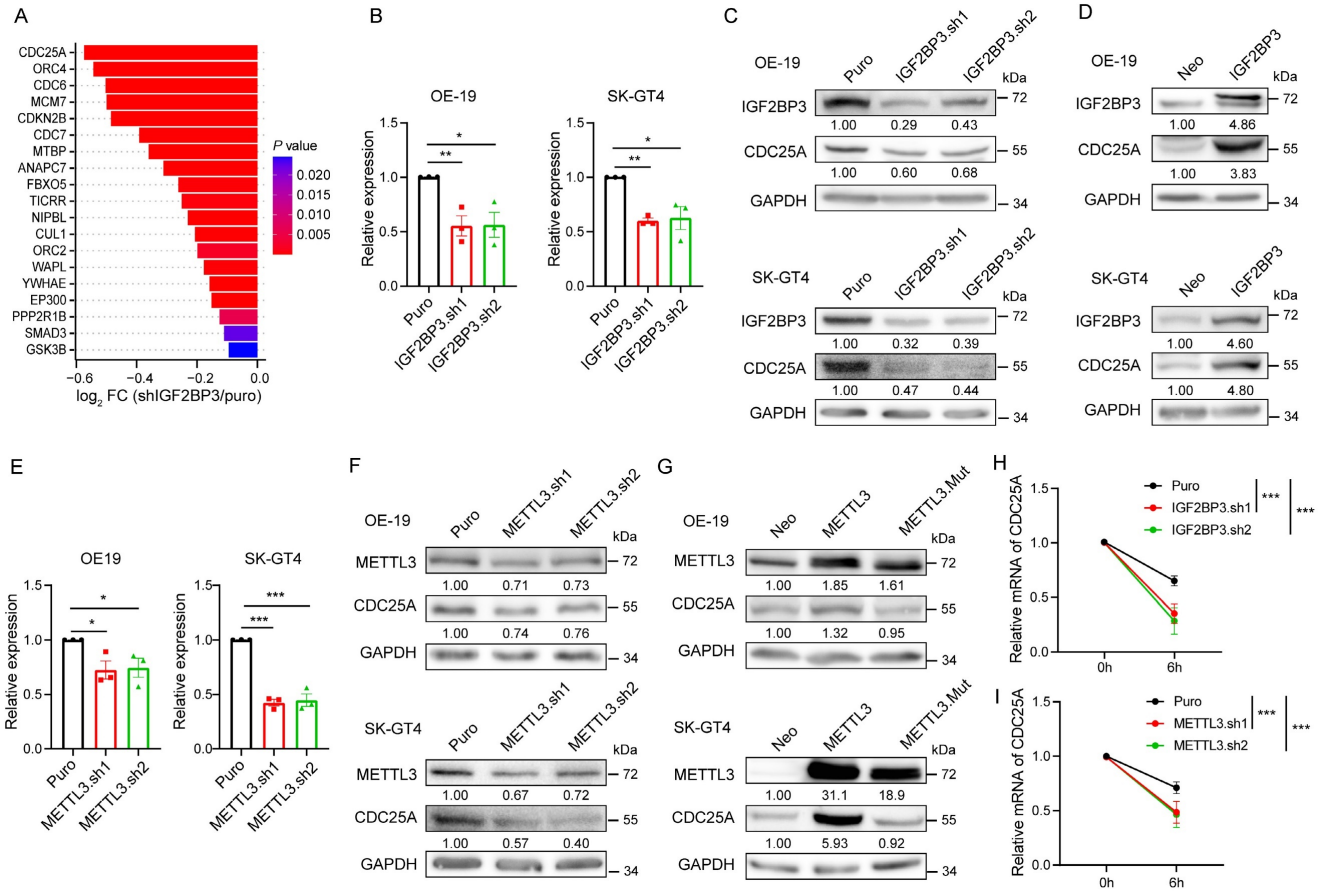
** IGF2BP3 stabilizes the *CDC25A* transcript in an m^6^A-dependent manner.** (A) Differential expression analysis of cell cycle related genes bound by IGF2BP3 in an m^6^A-dependent manner upon IGF2BP3 KD. (B) qRT-PCR analysis of *CDC25A* in IGF2BP3 KD OE-19 and SK-GT4 cells. (C-D) Immunoblot analysis of CDC25A in IGF2BP3 KD (C), and IGF2BP3 OE (D) OE-19 (top) and SK-GT4 (bottom) cells. (E) qRT-PCR analysis of *CDC25A* in METTL3 KD OE-19 and SK-GT4 cells. (F-G) Immunoblot analysis of CDC25A in METTL3 KD (F), and METTL3 OE (G) OE-19 and SK-GT4 cells. (H-I) Half-life of *CDC25A* after treatment with 5 μM actinomycin D for the indicated times in IGF2BP3 (H) or METTL3 (I) KD OE-19 cells. *p < 0.05, **p < 0.01, and ***p < 0.001.

To investigate whether and how IGF2BP3 affects the cell cycle of AEG cells in an m^6^A-dependent manner, we first performed an analysis for IGF2BP3 m^6^A-dependent-bound genes that are involved in the cell cycle and down-regulated in IGF2BP3 KD, and found that *CDC25A* was the most significantly down-regulated gene (Fig. 4A). Moreover, CDC25A has been shown to be a critical regulator of the G1-S transition of mammalian cells^34^, thus we focused on the CDC25A that as a potential m^6^A target of IGF2BP3. qRT-PCR and immunoblot assays showed that both mRNA and protein levels of CDC25A were significantly decreased in both IGF2BP3 KD OE-19 and SK-GT4 cells (Fig.4 B-C). In contrast, the OE of IGF2BP3 markedly increased CDC25A protein levels (Fig. 4D). We further assessed whether the methyltransferase METTL3 and demethylases ALKBH5/FTO are involved in the regulation of CDC25A expression. Strikingly, METTL3 KD down-regulated CDC25A expression (Fig. 4E-F), whereas the OE of WT METTL3, but not the catalytically inactive METTL3 mutant, markedly increased CDC25A expression levels (Fig. 4G). In contrast, ALKBH5 and FTO KD up-regulated CDC25A expression (Fig. S4A), whereas the OE of WT ALKBH5 and FTO, but not the catalytically inactive mutant, markedly decreased CDC25A expression levels (Fig. S4B). These results suggest that m^6^A regulates the expression of CDC25A.

To further determine how IGF2BP3 and METTL3 regulate CDC25A expression, we treated OE-19 cells with the transcriptional inhibitor actinomycin D and assessed the mRNA stability of *CDC25A*, and found that both IGF2BP3 and METTL3 KD significantly decreased the half-lives of *CDC25A* mRNA (Fig. 4H-I). Collectively, these data demonstrate that IGF2BP3 stabilizes the CDC25A transcript and increases its expression in an m^6^A-dependent manner.

### Identification of CDC25A m^6^A sites

**Figure 5:**
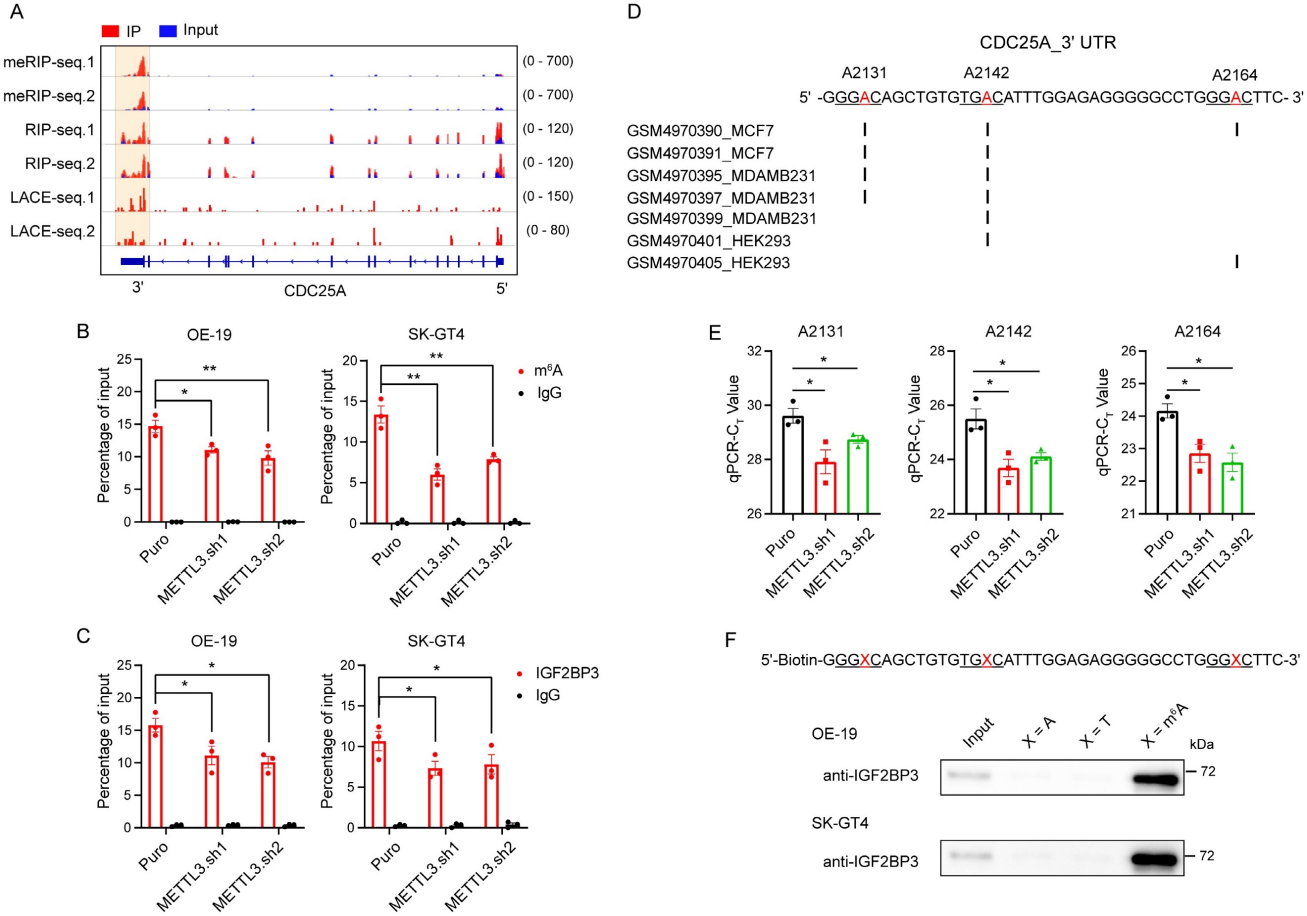
** Identification of CDC25A m^6^A sites.** (A) Distribution of peaks across the *CDC25A* transcript based on meRIP-seq, IGF2BP3 RIP-seq and LACE-seq data (n = 2). (B) m^6^A RIP-qPCR showing the enrichment of m^6^A modification in the *CDC25A* 3' UTR in METTL3 KD OE-19 (left) and SK-GT4 (right) cells. (C) RIP-qPCR showing the enrichment of IGF2BP3 in the *CDC25A* 3' UTR in METTL3 KD OE-19 (left) and SK-GT4 (right) cells. (D) CLIP-seq datasets reveal m^6^A modification sites in the *CDC25A* 3' UTR. (E) Threshold cycle (Ct) of qPCR showing SELECT results of A2131, A2142 and A2164 sites of *CDC25A* in METTL3 KD OE-19 cells. (F) Immunoblot detecting the enrichment of IGF2BP3 in RNA pulldown results using non-m^6^A or m^6^A *CDC25A* 3' UTR probes in OE-19 (top) and SK-GT4 (bottom) cells. *p < 0.05, **p < 0.01, and ***p < 0.001.

To determine how IGF2BP3 regulates CDC25A expression through m^6^A modification, we analyzed IGF2BP3 RIP-seq, LACE-seq, and meRIP-seq data. We identified that the m^6^A peaks, which coincide well with IGF2BP3-binding sites, are enriched in the 3′ UTR of the CDC25A transcript (Fig. 5A). To verify this finding, we performed m^6^A-RIP qPCR analysis and found significant m^6^A modifications in the 3′ UTR of *CDC25A* in OE-19 and SK-GT4 cells (Fig. S5A). These modifications were significantly decreased in METTL3 KD OE-19 and SK-GT4 cells (Fig. 5B). Consistent with these results, RIP qPCR analysis revealed that IGF2BP3 is remarkably enriched in the 3′ UTR of *CDC25A* (Fig. S5B), and these enrichments were significantly decreased in METTL3 KD OE-19 and SK-GT4 cells (Fig. 5C), suggesting that IGF2BP3 binds to the 3′ UTR of the *CDC25A* transcript in an m^6^A-dependent manner.

To further identify the m^6^A sites on the *CDC25A* 3′ UTR, we analyzed published single-nucleotide resolution crosslinking-immunoprecipitation followed by high-throughput sequencing datasets and identified three RRACH sites in the 3′ UTR of *CDC25A* that are potentially modified by m^6^A (Fig. 5D). Next, we verified the three potential methylation sites *in vivo* using the SELECT method achieving single-base resolution accuracy^11^, ^23^, and found that m^6^A levels at these sites significantly decreased in METTL3 KD OE-19 cells (Fig. 5E). To confirm IGF2BP3 binding to these m^6^A sites, we performed RNA pulldown using biotinylated and m^6^A-modified RNA probes. Immunoblot results showed specific IGF2BP3 enrichment in the m^6^A probe pulldown fraction, but not in non-m^6^A and mutant probes (Fig. 5F). Together, these data suggest that the *CDC25A* 3' UTR contains three m^6^A sites required for IGF2BP3 binding.

### IGF2BP3 increases CDC25A expression through three m^6^A sites in its 3' UTR

**Figure 6:**
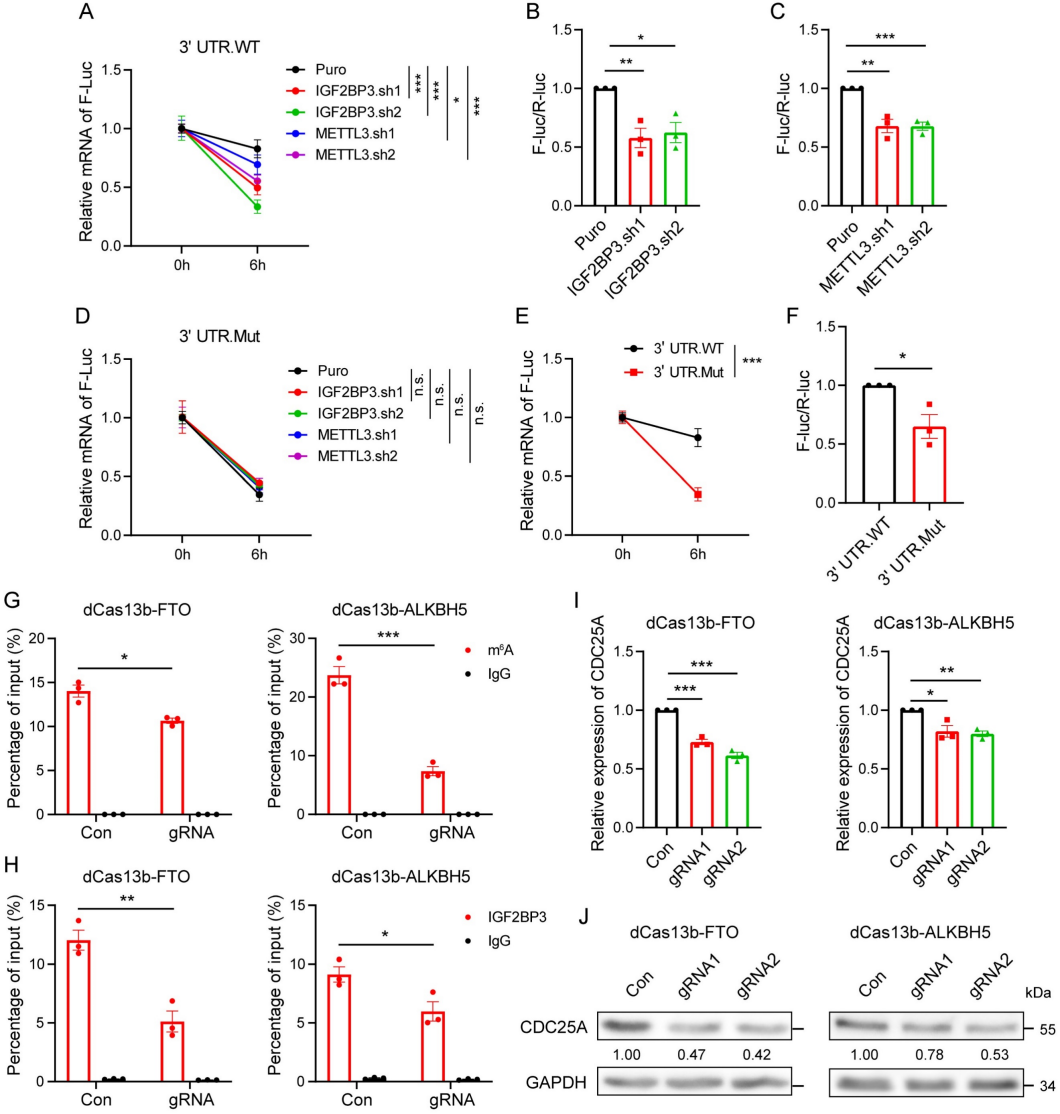
** IGF2BP3 increases CDC25A expression through three m^6^A sites in its 3' UTR.** (A and D) Half-lives of *F-Luc* coupling 3' UTR WT (A) and mutant (D) of *CDC25A* after treatment with 5 μM actinomycin D for the indicated times in IGF2BP3 and METTL3 KD 293T cells. (B and C) Dual-luciferase assays showing luciferase activities of *CDC25A* 3' UTR in IGF2BP3 or METTL3 KD 293T cells. (E) Half-lives of *F-Luc* coupling 3' UTR WT and mutant of *CDC25A* after treatment with 5 μM actinomycin D for the indicated times in 293T cells. (F) Dual-luciferase assays showing luciferase activities of the *CDC25A* 3' UTR WT and mutant in the 293T cells. (G-H) RIP-qPCR showing the enrichment of m^6^A modification (G) and IGF2BP3 (H) in the *CDC25A* 3' UTR in OE-19 cells transfected with dm^6^ACRISPR systems of FTO and ALKBH5. (I-J) qRT-PCR and immunoblot analysis of mRNA (I) and protein (J) expression of CDC25A in OE-19 cells transfected with dm^6^ACRISPR systems of FTO and ALKBH5. *p < 0.05, **p < 0.01, and ***p < 0.001.

To define the effect of the three m^6^A sites on CDC25A expression, we constructed a pmirGLO-*CDC25A* 3' UTR luciferase reporter (Fig. S6A). mRNA stability and dual-luciferase assays showed that IGF2BP3 and METTL3 KD significantly decreased the half-lives of *F-Luc* mRNA and luciferase activities of the *CDC25A* 3' UTR WT reporter (Fig. 6A-C). Subsequently, we designed reporters containing mutations (MUT) in the three m^6^A sites (Fig. S6A) and found that IGF2BP3 or METTL3 KD did not affect the half-lives of *F-luc* mRNA of the *CDC25A* 3' UTR MUT reporter (Fig. 6D). However, the mutations in the three m^6^A sites significantly reduced the half-lives of *F-luc* mRNA and luciferase activities of the reporter compared to the WT reporter (Fig. 6E-F). These results suggest that IGF2BP3 stabilizes the *CDC25A* transcript through three m^6^A sites in its 3' UTR.

We next specifically demethylated the m^6^A modification of *CDC25A* using the dm^6^ACRISPR-FTO/ALKBH5 system to confirm the effects of m^6^A on CDC25A expression. Two gRNAs targeting sequences around the three m^6^A sites were designed to target *CDC25A* (Fig. S6B). To test the efficiency of the gRNAs, we analyzed the mRNA levels of *CDC25A* in cells transfected with gRNAs and WT Cas13b. qRT-PCR analysis showed that the two gRNAs co-transfected with WT Cas13b, but not the transfection of gRNAs alone or combined with dCas13b, significantly decreased the mRNA level of *CDC25A* (Fig. S6C), suggesting that the two gRNAs can efficiently recognize *CDC25A*. Next, we verified the demethylation effects of the dm^6^ACRISPR system on *CDC25A*. m^6^A-RIP-qPCR analysis showed that both dCas13b-ALKBH5 and dCas13b-FTO combined with gRNAs decreased the m^6^A level of the *CDC25A* 3' UTR (Fig. 6G). RIP qPCR analysis showed that the binding ability of IGF2BP3 to *CDC25A* was significantly decreased after the m^6^A of *CDC25A* were demethylated by the dm^6^ACRISPR system (Fig. 6H). We then tested the effect of *CDC25A* 3′ UTR demethylation on its expression and found that dm^6^ACRISPR targeting *CDC25A* led to significant downregulation of *CDC25A* mRNA and protein levels in OE-19 cells (Fig. 6I-J). Together, these data suggest that the *CDC25A* 3' UTR contains three m^6^A sites required for IGF2BP3 binding and CDC25A expression.

### IGF2BP3 regulates cell cycle and cancer growth through CDC25A

**Figure 7:**
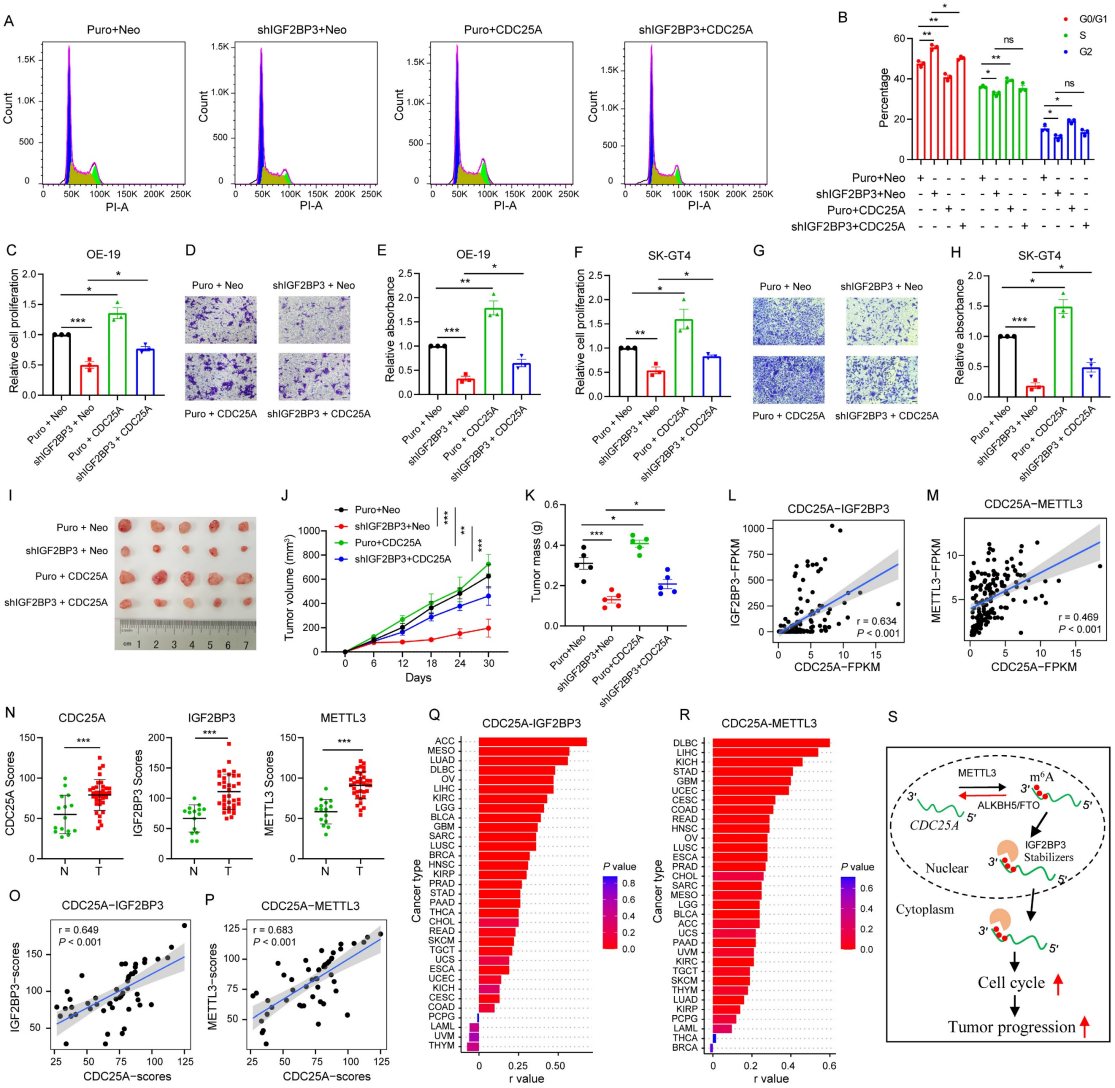
** IGF2BP3 regulates cell cycle and cancer growth through CDC25A.** (A and B) Flow cytometry analysis showing the percentage of IGF2BP3 KD with CDC25A OE in OE-19 cells in sub-G0-G1, S, and G2-M cell-cycle phases (n = 3). Representative cell-cycle profiles (A) and quantification analysis (B) are shown. (C-H) Cell proliferation and Transwell assays assessing IGF2BP3 KD with CDC25A OE in OE-19 (C-E) and SK-GT4 (F-H) cells. (I-K) Effects of IGF2BP3 KD with CDC25A OE in OE-19 cells on tumor size (I), tumor growth (J) and mass (K) in nude mice (n = 5). (L-M) Correlation between* CDC25A* and *IGF2BP3* (L) or *METTL3* (M) in 83 AEG patients. (N-P) IHC analysis of CDC25A, IGF2BP3 and METTL3 in AEG tumor tissues. The quantification analysis is shown (N). Spearman correlation analysis between CDC25A and IGF2BP3 (O) or METTL3 (P). Normal adjacent tissues (N) = 15; Tumor tissues (T) = 30. (Q-R) Spearman correlation between* CDC25A* and *IGF2BP3* (Q) or *METTL3* (R) across 33 cancer types in TCGA datasets. (S) Proposed model for the METTL3/IGF2BP3/CDC25A axis regulates cancer cell growth. Scale bars, 20 µm. *p < 0.05, **p < 0.01, and ***p < 0.001.

Given these findings, we analyzed whether IGF2BP3 regulates the cell cycle and cancer growth through CDC25A by performing rescue experiments. Flow cytometry assays showed that CDC25A OE prevented the decreased G1-S transition in the cell cycle induced by IGF2BP3 KD (Fig. 7A-B). Furthermore, CDC25A OE partially reversed the reduction in cell proliferation and migration caused by IGF2BP3 KD in OE-19 and SK-GT4 cells (Fig. 7C-H). To further confirm the effects of the IGF2BP3/CDC25A axis on AEG progression, we performed *in vivo* xenograft experiments and found that CDC25A OE partially reversed the tumor growth reduction caused by IGF2BP3 KD (Fig. 7I-K), suggesting that CDC25A is involved in IGF2BP3-mediated cell cycle and cancer growth in AEG.

To further determine the clinical relevance of the METTL3/IGF2BP3/CDC25A axis, we analyzed the RNA correlation of these genes in RNA-seq data from 83 AEG patients^6^. Results showed that *CDC25A* had a strong positive correlation with both *IGF2BP3* (r = 0.634, *P* < 0.001) and *METTL3* (r = 0.469, *P* < 0.001) (Fig. 7L-M). Next, we performed IHC staining in 30 AEG tumors, and 15 paired adjacent normal tissues. IHC results showed that METTL3, IGF2BP3, and CDC25A expression significantly increased in cancer tissues compared to normal adjacent tissues (Fig. 7N, and Fig. S7). In particular, a strong positive correlation was observed between IGF2BP3 and CDC25A expression (r = 0.649, *P* < 0.001), and between METTL3 and CDC25A expression (r = 0.683, *P* < 0.001) (Fig. 7O-P). More importantly, *CDC25A* expression was positively correlated with *IGF2BP3* and *METTL3* expression in most cancer types in TCGA datasets (Fig. 7Q-R). These results support that CDC25A is clinically associated with METTL3 and IGF2BP3 in AEG cancer.

## Discussion

The genomic, transcriptomic, proteomic, and phosphoproteomic profiling of large cohort of AEG patients had been studied, here, our study provides the post-transcriptional regulation of AEG. By performing a transcriptome-wide m^6^A methylome analysis in AEG, we found that m^6^A modification plays a key role in regulating AEG tumor progression. Specifically, IGF2BP3 mediates m^6^A modification on the expression of critical genes, such as *CDC25A*. By positively regulating the mRNA stability of these transcripts, IGF2BP3 promotes the cell cycle, allowing for the rapid proliferation and survival of AEG cells (Fig. 7S).

Among m^6^A regulators, METTL3/14, FTO, ALKBH5, and members of the YTHDF family have been extensively studied for their contributions to RNA regulation and cancer progression^35^-^38^. We found that IGF2BP3 is the most up-regulated m^6^A regulator in AEG tumors, suggesting that IGF2BP3 may play a major role in AEG. Recent studies have reported that IGF2BP3 binds to certain target genes, such as *CDK4*^21^, *CCND1*^39^, and *MYC* 40, in an m^6^A-dependent manner to govern cell cycle progression. In this study, we identified a broad spectrum of target genes regulated by IGF2BP3 in the cell cycle, further elucidating the significant role of IGF2BP3 in regulating the cell cycle. Importantly, silencing IGF2BP3 results in a significant decrease in AEG progression *in vitro* and *in vivo*, highlighting a therapeutic window for targeting IGF2BP3 in AEG treatment.

The cell cycle plays a crucial role in cancer development and progression, and aberrant cell cycle regulation is a hallmark of cancer^41^. CDC25A is a phosphatase enzyme that activates cyclin-dependent kinases by removing inhibitory phosphate groups^42^. This activation leads to cell cycle progression from the G1 to the S phase^34^. Overexpression of CDC25A has been frequently documented in multiple cancer cell lines, and is highly associated with malignancy and poor prognosis in cancer patients^43^, ^44^. Several mechanisms regulate the expression and activity of CDC25A, including transcriptional^45^, ^46^, post-transcriptional^47^, ^48^, and post-translational^49^, ^50^ regulation. However, the post-transcriptional modification and regulation of the CDC25A gene remain unclear. Here, we identified three m^6^A sites in the *CDC25A* 3' UTR, that are required for IGF2BP3 binding and CDC25A expression. Importantly, these specific m^6^A modification sites of *CDC25A*, associated with its expression, could be promising therapeutic targets for CDC25A-associated malignancies.

## Supplementary Material

Supplementary figures and tables.
