# Supplementary material for: m6A modification enhances the stability of CDC25A promotes tumorigenicity of esophagogastric junction adenocarcinoma via cell cycle
Source: Int J Biol Sci. 2024 Aug 6;20(11):4209–21. doi: 10.7150/ijbs.98535 (PMC11379066; doi:10.7150/ijbs.98535)
Supplement: Supplementary file 1 — Supplementary figures and tables. [file ijbsv20p4209s1.pdf]

Supplementary Information

m^{6}A modification enhances the stability of *CDC25A* promotes tumorigenicity of esophagogastric junction adenocarcinoma via cell cycle

Yongbo Pan[a,b,c,1], Huolun Feng[b,1], Jianlong Zhou[b,1], Wenxing Zhang[b], Yongfeng Liu[b], Jiabin Zheng[b], Junjiang Wang[b], Shan Gao[c,2], Yong Li[b,2]

[a]Guangdong Cardiovascular Institute, Guangdong Provincial People's Hospital, Guangdong Academy of Medical Sciences, Guangzhou 510080, China

[b]Department of Gastrointestinal Surgery, Department of General Surgery, Guangdong Provincial People's Hospital (Guangdong Academy of Medical Sciences), Southern Medical University, Guangzhou 510080, China

[c]Zhongda Hospital, School of Life Sciences and Technology, Advanced Institute for Life and Health, Southeast University, Nanjing 210096, China

[1]Y.P., H.F. and J.Z. contributed equally to this work.

[2]To whom correspondence may be addressed. Email: gaos@sibet.ac.cn and liyong@gdph.org.cn

This supplementary information contains:

- 15 Pages
- Supplementary Figures (7 Figures)
- Supplementary Tables (6 Tables)

Supplementary Figures

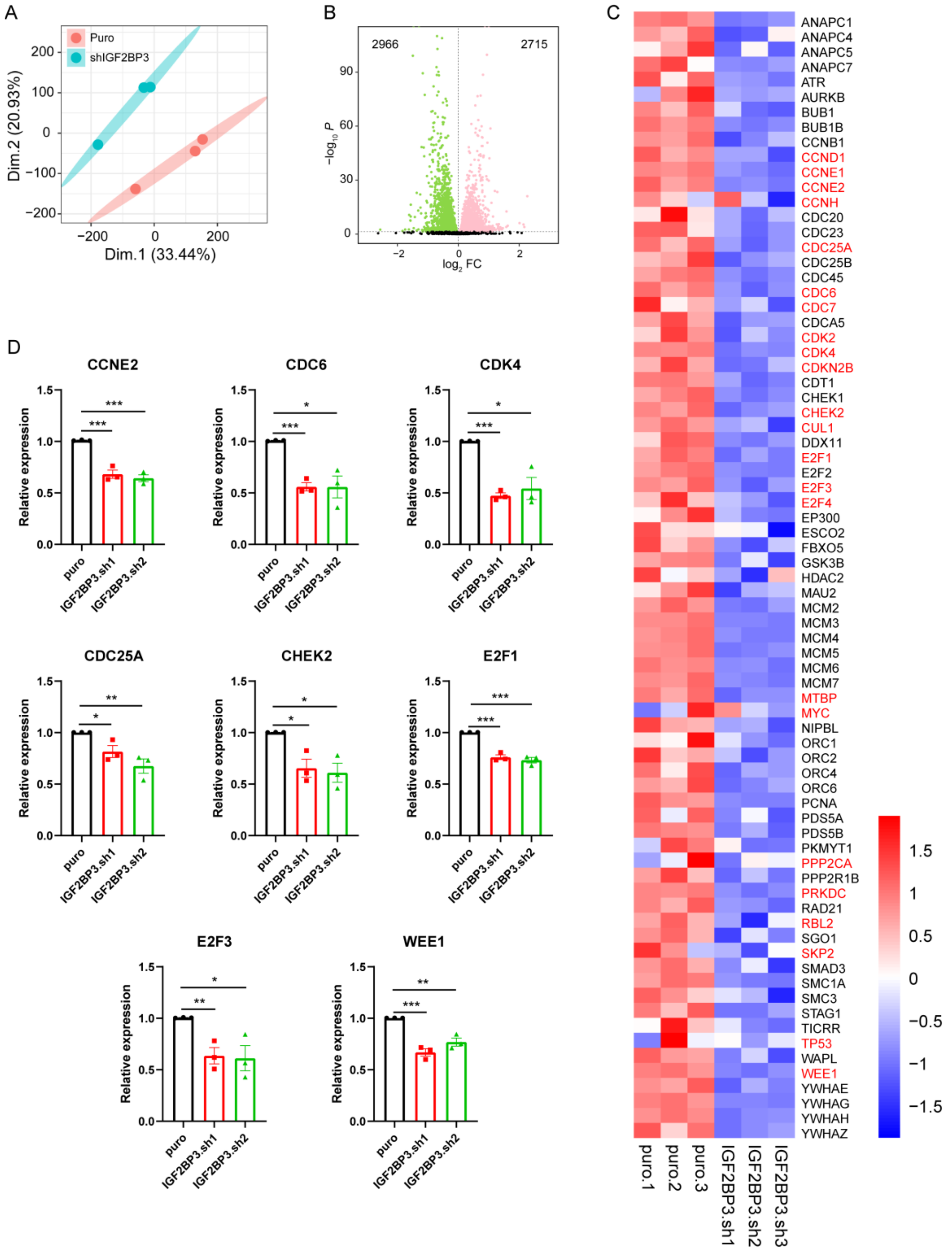

Fig. S1 | RNA-seq analysis of IGF2BP3 KD OE-19 cells. (A) Principal component analysis plot of RNA-seq data for Puro and shIGF2BP3 groups. (B) Volcano plot showing the differentially expressed genes (DEGs) in IGF2BP3 KD OE-19 cells. (C) Heatmap showing 75 cell cycle-related and down-regulated genes in IGF2BP3 KD OE-

19 cells. The red marks indicate G1-S transition-regulated genes. (D) qPCR analysis of the mRNA expression levels of 8 G1-S transition-regulated genes in IGF2BP3 KD OE-19 cells.

Related to Fig. 2.

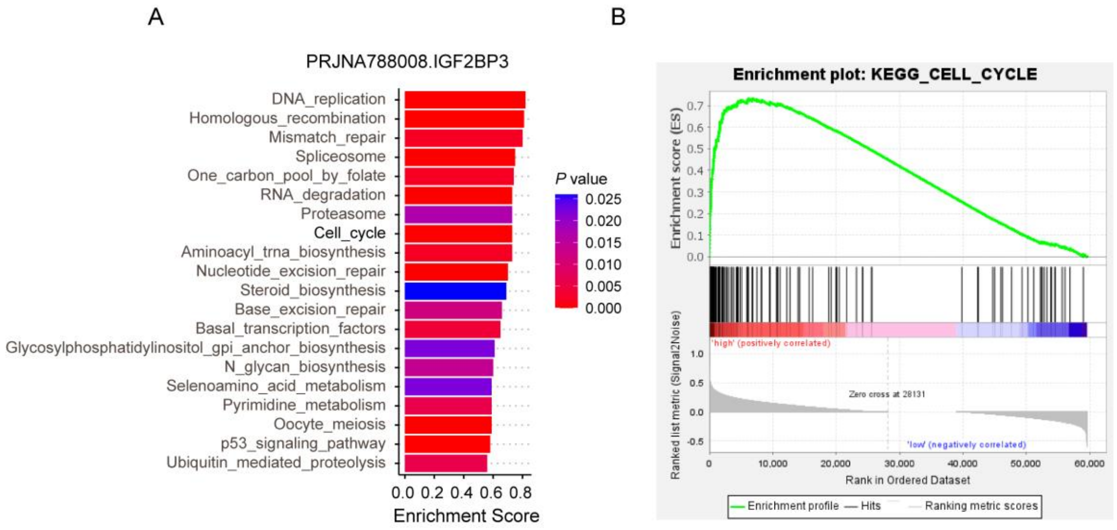

Fig. S2 | IGF2BP3 regulates the cell cycle pathway in AEG. (A-B) Gene set enrichment analysis (GSEA) showing the top 20 significantly enriched signaling pathways (A), and the “Cell cycle” signaling pathways (B) in the RNA-seq data of 83 AEG patients based on the median value of IGF2BP3 expression.

Related to Fig. 2.

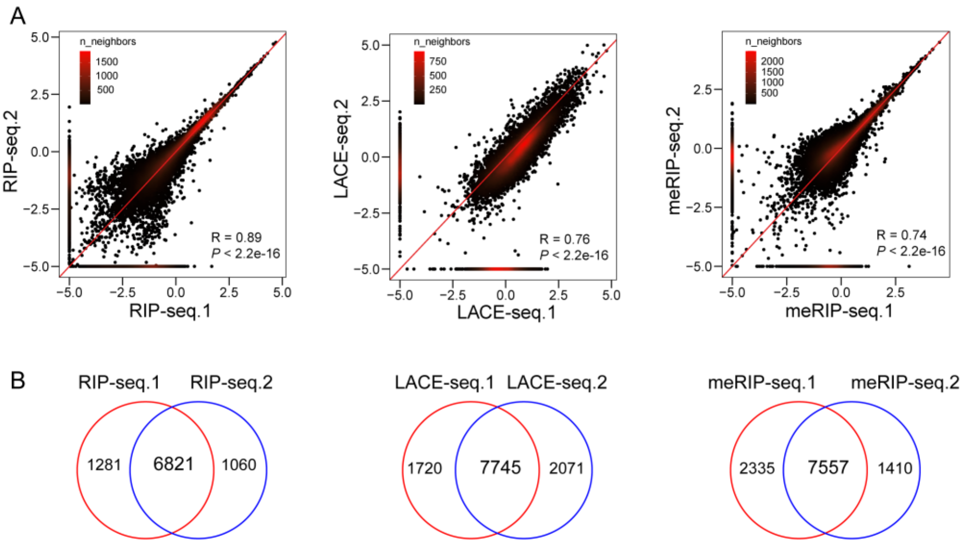

Fig. S3 | RIP-seq, LACE-seq and meRIP-seq analysis of IGF2BP3 in AEG. (A) Scatterplot showing the high reproducibility of the RIP-seq, LACE-seq, and meRIP-seq replicates. The Pearson correlation coefficients (R) of the normalized reads across the two replicates were calculated and displayed in the plots. A smoother regression line and 2D kernel density contour bands are also presented. *P* values were determined by Pearson's correlation test. (B) Overlap of IGF2BP3 target genes identified by RIP-seq and LACE-seq, and m^6A modified genes by meRIP-seq in OE-19 cells.

Related to Fig. 3.

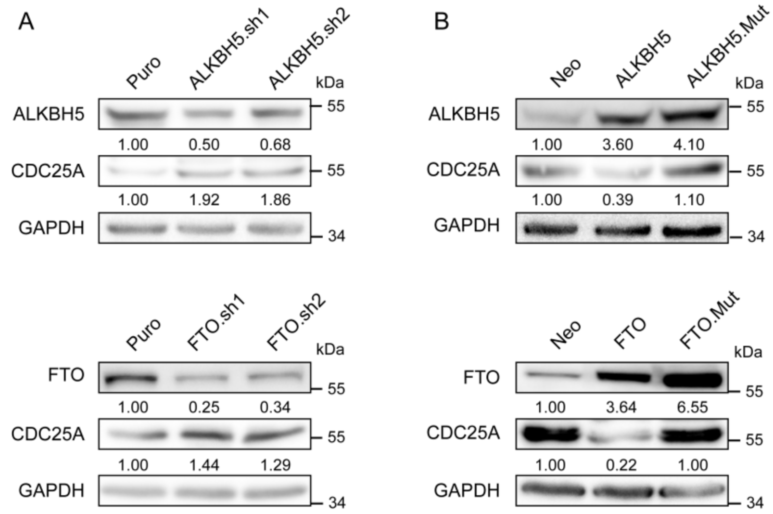

Fig. S4 | ALKBH5 and FTO regulate CDC25A expression. (A) Immunoblot analysis of CDC25A in ALKBH5 and FTO KD OE-19 cells. (B) Immunoblot analysis of CDC25A in ALKBH5 and FTO OE OE-19 cells.

Related to Fig. 4.

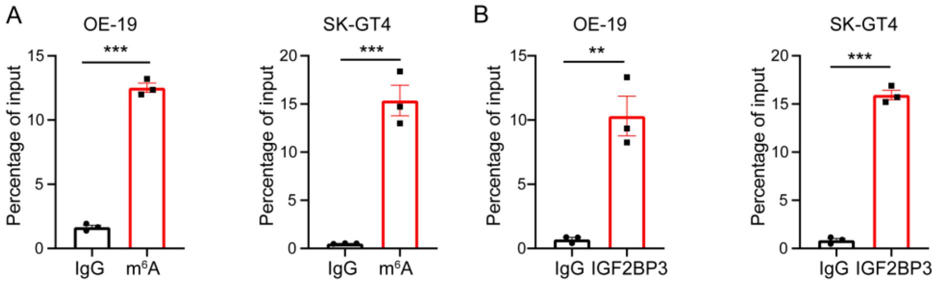

Fig. S5 | The enrichment of m^{6}A and IGF2BP3 in the *CDC25A* 3' UTR. (A) m^{6}A RIP-qPCR showing the enrichment of m^{6}A modification in the *CDC25A* 3' UTR OE-19 and SK-GT4 cells. (B) RIP-qPCR showing the enrichment of IGF2BP3 in the *CDC25A* 3' UTR in OE-19 and SK-GT4 cells.

Related to Fig. 5.

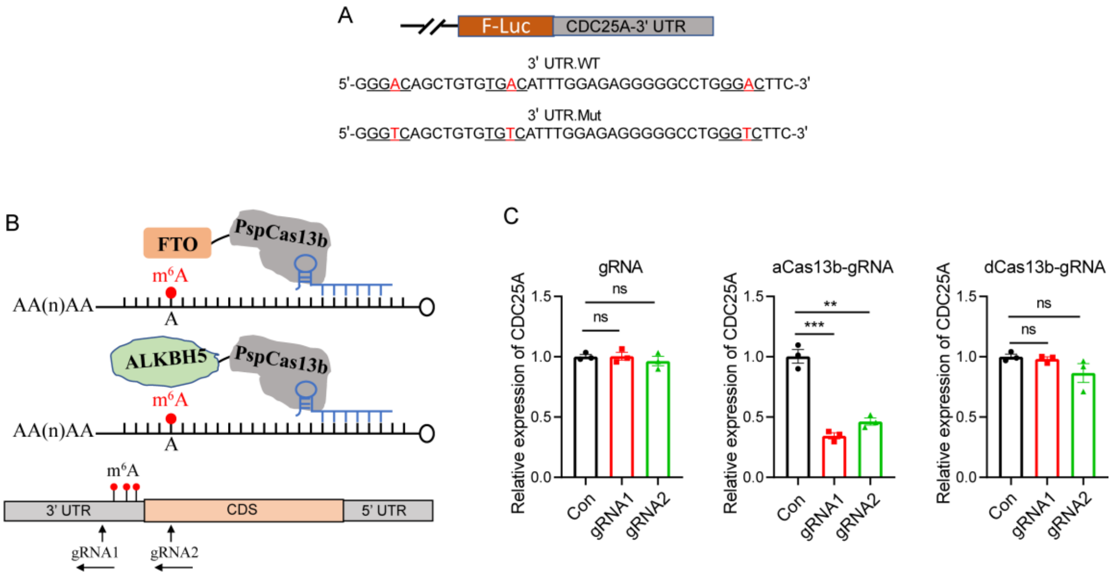

Fig. S6 | The dm⁶ACRISPR-FTO/ALKBH5 system targets the *CDC25A* 3' UTR. (A) Schematic representation of mutation sites of pmirGLO-*CDC25A* 3' UTR luciferase reporter. (B) Overview of site-specific RNA targeting of *CDC25A* using dCas13b-guided fusion proteins with two gRNAs. (C) qRT-PCR analysis of *CDC25A* expression in OE-19 cells transfected with gRNAs alone (left), gRNAs combined with Cas13b (middle) or dCas13b (right).

Related to Fig. 6.

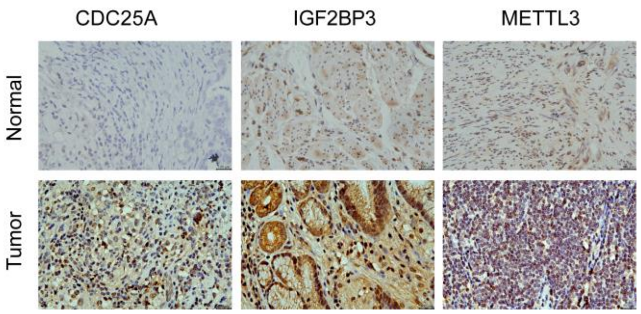

Fig. S7 | IHC analysis of CDC25A, IGF2BP3, and METTL3 in AEG tumor tissue microarray. Representative images of tissue sections are shown. Normal adjacent tissues (N) = 15; Tumor tissues (T) = 30. Scale bars, 100 µm.

Related to Fig. 7.

Supplementary Tables

Table S1 List of patient analysis in this study.

ID	Gender	Age	AEG tissue	With adjacent normal tissue	qRT-PCR immunoblot	IHC
T1	male	73	√	√	√	√
T2	male	64	√	√	√	√
T3	male	55	√	√	√	√
T4	female	77	√	√	√	√
T5	male	77	√	√	√	√
T6	male	82	√	√		√
T7	male	68	√	√		√
T8	female	52	√	√		√
T9	male	63	√	√		√
T10	male	65	√	√		√
T11	male	54	√	√		√
T12	male	55	√	√		√
T13	male	65	√	√		√
T14	male	70	√	√		√
T15	male	63	√	√		√
T16	male	50	√			√
T17	male	55	√			√
T18	male	81	√			√
T19	male	60	√			√
T20	male	61	√			√
T21	male	62	√			√
T22	male	46	√			√
T23	female	60	√			√
T24	male	55	√			√
T25	female	59	√			√
T26	male	68	√			√
T27	male	69	√			√
T28	male	80	√			√
T29	female	71	√			√
T30	male	42	√			√

Table S2 Sequences of shRNA for KD.

	**Forwards (5'-3')**
IGF2BP3.sh1	GCAAAGGATTCGGAAACTTCA
IGF2BP3.sh2	GCTGAGAAGTCGATTACTATC
METTL3.sh1	GCTGCACTTCAGACGAATTAT
METTL3.sh2	GGATACCTGCAAGTATGTTCA
ALKBH5.sh1	TCCTTGTCCATCTCCAGGATC
ALKBH5.sh2	TATGCAGTGAGTGATTTCATC
FTO.sh1	TGAACCTCTTTATGGAGCTCC
FTO.sh2	ACATTCTGGCTTCTGATCAGC

Table S3 List of qRT-PCR primers.

	**Forwards (5'-3')**	**Reverses (5'-3')**
IGF2BP3	TCGAGGCGCTTTCAGGTAAA	AAACTATCCAGCACCTCCCAC
METTL3	ATTTTCCGGTTAGCCTTCGGG	CATCCTAGTCTCCCAGCCCT
SELECT	TAGCCAGTACCGTAGTGCGTG	ATGCAGCGACTCAGCCTCTG
GAPDH	GGAGCGAGATCCCTCCAAAAT	GGCTGTTGTCATACTTCTCATGG
CDC25A	GTGGGAGAACAGCGAAGACA	AATCCAAACAAACGTGGCGG
CDC25A 3' UTR	CAAAGGGGACAGCTGTGTGA	GACAGAAGAGGCGTAGCCAG
CCNE2	TAGCTGGTCTGGCGAGGT	GGGCTGCTGCTTAGCTTGTA
CDC6	GCGAGGCCTGAGCTGTG	GCTGAGAGGCAGGGCTTTTA
CDK4	GGCCTGTGTCTATGGTCGG	GGCACCGACACCAATTTCAG
CHEK2	GAGAGTGTGCGGCTCCAG	GAGCCTTGGGACTGGGTAAC
E2F1	TCGTAGCATTGCAGACCCTG	ACATCGATCGGGCCTTGTTT
E2F3	CAGGCTGGTTTCGGAAATGC	TGGACTTCGTAGTGCAGCTC
WEE1	CTGAACAATGGGCCTCGTCT	ATCCTATGGCTCGGGAGTGT

Table S4 List of primary antibodies.

Antibodies	Manufacturer	Application
GAPDH	Proteintech, #60004-1-Ig	1:3000 for WB
IGF2BP3	Proteintech, #14642-1-AP	1:3000 for WB, 3 µg for RIP, 1:400 for IHC
METTL3	Proteintech, #15073-1-AP	1:3000 for WB, 1:400 for IHC
CDC25A	Proteintech, #55031-1-AP	1:3000 for WB, 1:300 for IHC
ALKBH5	Proteintech, #16837-1-AP	1:2000 for WB
FTO	Proteintech, #27226-1-AP	1:2000 for WB
m^{6}A	Synaptic Systems, #202003	3 µg for RIP-qPCR

Table S5 Primers for SELECT qPCR.

	Sequence (5'-3')
A2131.up	tagccagtaccgtagtgcgtgCCTCTCCAAATGTCACACAGCTG
A2131.down	5phos/CCCCTTTGCTTAAGTTTCTCTGcagaggctgagtcgctgcat
A2142.up	tagccagtaccgtagtgcgtgGTCCCAGGCCCCCTCTCCAAATG
A2142.down	5phos/CACACAGCTGTCCCCTTTGCTTAcagaggctgagtcgctgcat
A2164.up	tagccagtaccgtagtgcgtgGAGGTAGGTTTAAGGCATGGAAG
A2164.down	5phos/CCCAGGCCCCCTCTCCAAATGTCcagaggctgagtcgctgcat
N.up	tagccagtaccgtagtgcgtgGGCATGGAAGTCCCAGGCCCCCTC
N.down	5phos/CCAAATGTCACACAGCTGTCCCcagaggctgagtcgctgcat

86 **Table S6 G1-S transition-regulating genes and supporting references.**

Gene	Reference
CCND1	Pedraza N et al. Cyclin D1-Cdk4 regulates neuronal activity through phosphorylation of GABAA receptors. CELL MOL LIFE SCI: CMLS 2023;80:280
CCNE1	Sonntag R, et al. Cyclin E1 and cyclin-dependent kinase 2 are critical for initiation, but not for progression of hepatocellular carcinoma. P NATL ACAD SCI USA 2018;115:9282-7
CCNE2	Sonntag R, et al. Cyclin E1 and cyclin-dependent kinase 2 are critical for initiation, but not for progression of hepatocellular carcinoma. P NATL ACAD SCI USA 2018;115:9282-7
CCNH	Hume S, et al. A unified model for the G1/S cell cycle transition. Nucleic Acids Res 2020;48:12483-501
CDC25A	Sur S, et al. Phosphatases and kinases regulating CDC25 activity in the cell cycle: clinical implications of CDC25 overexpression and potential treatment strategies. MOL CELL BIOCHEM 2016;416:33-46
CDC6	Oehlmann M, et al. The role of Cdc6 in ensuring complete genome licensing and S phase checkpoint activation. JCB 2004;165:181-90
CDC7	Moiseeva TN, et al. WEE1 kinase inhibitor AZD1775 induces CDK1 kinase-dependent origin firing in unperturbed G1- and S-phase cells. P NATL ACAD SCI USA 2019;116:23891-3
CDK2	Sonntag R, et al. Cyclin E1 and cyclin-dependent kinase 2 are critical for initiation, but not for progression of hepatocellular carcinoma. P NATL ACAD SCI USA 2018;115:9282-7
CDK4	Pedraza N, et al. Cyclin D1-Cdk4 regulates neuronal activity through phosphorylation of GABAA receptors. CELL MOL LIFE SCI: CMLS 2023;80:280
CDKN2B	Arya AK, et al. Promoter hypermethylation inactivates CDKN2A, CDKN2B and RASSF1A genes in sporadic parathyroid adenomas. SCI REP 2017;7:3123
CHEK2	Zannini L, et al. CHK2 kinase in the DNA damage response and beyond. JCB 2014;6:442-57
CUL1	O'Hagan RC, et al. Myc-enhanced expression of Cul1 promotes ubiquitin-dependent proteolysis and cell cycle progression. GENE DEV 2000;14:2185-91
E2F1	Inoshita S, et al. Regulation of the G1/S transition phase in mesangial cells by E2F1.KIDNEY INT 1999;56:1238-41
E2F3	Inoshita S, et al. Regulation of the G1/S transition phase in mesangial cells by E2F1.KIDNEY INT 1999;56:1238-41
E2F4	Inoshita S, et al. Regulation of the G1/S transition phase in mesangial cells by E2F1.KIDNEY INT 1999;56:1238-41
MTBP	Chirackal Manavalan AP, et al. CDK12 controls G1/S progression by regulating RNAPII processivity at core DNA replication genes. EMBO REP 2019;20:e47592
MYC	Liu J-Y, et al. LncRNA SNHG17 interacts with LRPPRC to stabilize c-Myc protein and promote G1/S transition and cell proliferation. CELL DEATH DIS 2021;12:970
PPP2CA	Yan Y, et al. Distinct roles for PP1 and PP2A in phosphorylation of the retinoblastoma protein. PP2a regulates the activities of G(1) cyclin-dependent kinases. JBC 1999;274:31917-24
PRKDC	Blackford AN, et al. The Trinity at the Heart of the DNA Damage Response. Mol Cell 2017;66:801-17
RBL2	Militi S, et al. RBL2-E2F-GCN5 guide cell fate decisions during tissue specification by regulating cell-cycle-dependent fluctuations of non-cell-autonomous signaling. Cell Rep 2023;42:113146
SKP2	Hume S, et al. The NUCKS1-SKP2-p21/p27 axis controls S phase entry. Nat Commun 2021;12:6959
TP53	Zannini L, et al. CHK2 kinase in the DNA damage response and beyond. J MOL CELL BIOL 2014;6:442-57
WEE1	Moiseeva TN, et al. WEE1 kinase inhibitor AZD1775 induces CDK1 kinase-dependent origin firing in unperturbed G1- and S-phase cells. P NATL ACAD SCI USA 2019;116:23891-3

87 **Related to Fig. S1.**
